# Relationship Between Case Volume and Outcomes for Pulmonary Resection for Lung Cancer at Safety-Net Hospitals

**DOI:** 10.1016/j.atssr.2025.11.015

**Published:** 2025-12-09

**Authors:** Carlin Lee, Sara Sakowitz, Avital Zucker, Kelly Fairbairn, Ali Mahtabifard, Peyman Benharash, Hari B. Keshava

**Affiliations:** 1 Division of Thoracic Surgery, Department of Surgery, University of California, Irvine, California; 2 Cardiac Outcomes Research Laboratory (CORELAB), Division of Cardiac Surgery, Department of Surgery, University of California, Los Angeles, California

## Abstract

**Background:**

Safety-net hospital (SNH) status is associated with high perioperative morbidity in pulmonary resection. Multiple etiologies have been proposed, including delays in diagnosis and inefficient care pathways. In integrated health systems, surgical volume has been shown to improve outcomes in pulmonary resection. However, whether surgical volume can overcome the inherent challenges of SNHs is unclear. We hypothesize that surgical volume is associated with improved outcomes at SNHs.

**Methods:**

The 2016 to 2021 Nationwide Readmissions Database was queried for all adult (≥18 years) patients undergoing elective lobectomy for lung cancer. Centers in the top quartile of Medicaid or self-pay/uninsured admissions were defined as SNHs. SNHs were further stratified by lobectomy caseload as a low-volume hospital (<10 cases/y), medium-volume hospital (10-33 cases/y), or high-volume hospital (>33 cases/y). Multivariable regressions were built to consider the independent association of hospital volume on acute clinical and financial outcomes among patients treated at SNHs.

**Results:**

Care at high-volume centers remained associated with significantly reduced likelihood of overall major morbidity (adjusted odds ratio [AOR], 0.81; 95% CI, 0.68-0.97), respiratory complications (AOR, 0.79; 95% CI, 0.65-0.96), need for blood transfusion (AOR, 0.67; 95% CI, 0.48-0.93), and nonhome discharge (AOR, 0.66; 95% CI, 0.48-0.88). Care at high-volume centers was also associated with a decrease in duration of hospitalization (β = −1.02 days; 95% CI, −1.48 to −0.54 days) and overall expenditures (β = −$4360; 95% CI, −$7020 to −$1700).

**Conclusions:**

Surgical volume is associated with improved outcomes in pulmonary resection at SNHs. Patients who are eligible for care only at SNHs can still benefit from undergoing pulmonary resection at a high-volume center.

**In Short**

▪ Lung cancer resection at safety-net hospitals was associated with an increased rate of complications.
▪ Lung cancer resection at safety-net hospitals was also associated with increased costs and expenditures.

Safety-net hospitals (SNHs) are defined by their common mission to provide care regardless of patient income or insurance status. They typically have access to fewer resources and serve a larger proportion of younger patients with lower socioeconomic status and more advanced illness.^1^ Given the large burden of underinsured patients at these institutions, a broad evaluation of complications, resource utilization, and cost for surgical procedures is important to identify systemic factors that may contribute to health inequity.

Recent studies have found that safety-net status is an independent risk factor for inferior surgical outcomes, which include mortality, serious complications, greater cost, and inadequate pain control.^2^ The established discrepancy between SNH and non-SNH systems has been in part attributed to limited resources and high penalty costs, leading to minimal investment in quality improvement.^3^

Elective pulmonary resection is a complex procedure, necessitating multidisciplinary involvement throughout the preoperative and postoperative stages.^4^ The challenges often faced by SNHs, such as access to resources and system inefficiency, may therefore be relevant to successful outcomes. In a recent study, Sakowitz and colleagues^4^ used the Nationwide Readmissions Database to analyze outcomes for elective lobectomy in SNHs over the span of a decade. Although mortality and rates of readmission were comparable, the study found higher odds of perioperative infection, prolonged mechanical ventilation, and cerebrovascular events.^4^

Many patients do not have coverage for elective surgery outside of the SNH system. For this population, identifying metrics that predict perioperative morbidity and mortality would better inform their decisions about center selection. In integrated health systems, high volume has been found to achieve lower rates of adverse events after a range of surgical procedures, including pulmonary resection.^5^ Given these findings, we hypothesized that higher volume may have the potential to compensate for systemic issues within SNHs. In our study, we examined the association between surgical volume and outcomes at SNHs for elective pulmonary resection for cancer.

## Material and Methods

The 2016 to 2021 Nationwide Readmissions Database (NRD) was queried for all adult (≥18 years) hospitalization records entailing elective lobectomy for lung cancer using validated International Classification of Diseases, 10th Revision (ICD-10) codes.^6^ Patients who underwent lobectomy for other reasons were not considered. This study was deemed exempt from full review by the University of California, Los Angeles and University of California, Irvine Institutional Review Boards.

The proportion of Medicaid or self-pay/uninsured admissions was computed for each institution, with centers in the top quartile defined as SNHs as previously described.^4^ Annual lobectomy caseload was used to further stratified SNHs into tertiles as low-volume hospital (LVH, <10 cases/y), medium-volume hospital (MVH, 10-33 cases/y), or high-volume hospital (HVH, >33 cases/y).

Patient and hospital characteristics were defined using the Healthcare Cost and Utilization Project data dictionary.^6^ The van Walraven medication of the Elixhauser Comorbidity Index was used to assess overall burden of comorbidities. Complications were identified using ICD-10 codes. Costs were computed using institution cost-to-charge ratios within the NRD and subsequently inflation-adjusted using the 2021 Personal Healthcare Price Index.

The primary outcome was mortality. Secondary outcomes included any major perioperative complication, including acute kidney injury, blood transfusion, cardiac complications, infectious complications, intraoperative complications, and respiratory complications. We also considered nonhome discharge and nonelective 30-day readmissions.

Continuous variables are reported as medians with interquartile ranges (IQRs) and categorical variables are reported as the percentage. A Mann-Whitney *U* test was used to compare continuous variables, and χ^2^ was used to compare categorical variables. Multivariable regressions were built to consider the independent association of hospital volume on acute clinical and financial outcomes among patients treated at SNHs. Model covariates were selected using elastic net regularization, an automated method that improves out-of-sample generalizability while optimizing model fit. Outcomes were reported as adjusted odds ratios (AOR) if logistic or as β-coefficients if linear, both with 95% CIs.

Statistical significance was defined as α = 0.05. All statistical analyses were performed using Stata 18.0 software (StataCorp LLC).

## Results

### Patient Demographic and Hospital Characteristics

Patient and hospital characteristics are featured in Table 1. Of a total 381 SNHs, 23% were considered LVH and 12% HVH. On average, and relative to patients at LVHs, those treated at HVHs were of similar sex (55.0% vs 53.0%, *P* = .41) but of a lower Elixhauser comorbidity score (3 [2-5] vs 4 [3-5], *P* < .001) and were less commonly insured by Medicaid (9.6% vs 20.6%, *P* < .001). Further, HVH patients more frequently received video-assisted thoracoscopic (41.5% vs 29.3%, *P* < .001) or robotic lobectomy (27.8% vs 6.9%, *P* < .001), relative to LVH patients (Table 1).

**Table 1:** Demographic, Clinical, and Hospital Characteristics During the Initial Admission

Variable	LVH	MVH	HVH	*P* Value
	(n = 2105)	(n = 6523)	(n = 19,300)	
Age, y	66 (61-73)	68 (62-73)	68 (61-74)	<.001
Female sex	53.0	54.6	55.0	.41
Elixhauser Comorbidity Index	4 (3-5)	4 (2-5)	3 (2-5)	<.001
Smoking status	70.2	74.1	73.1	.14
Approach				<.001
Open	63.8	47.1	30.7	
VATS	29.3	32.8	41.5	
RATS	6.9	20.1	27.8	
Income quartile				.02
>75%	15.8	19.5	20.2	
51%-75%	22.6	24.6	24.7	
26%-50%	23.0	25.6	26.9	
0%-25%	38.6	30.3	28.2	
Insurance coverage				<.001
Private	17.7	18.4	24.2	
Medicare	56.6	63.9	61.8	
Medicaid	20.6	13.8	9.6	
Self-pay	1.6	1.3	1.3	
Other payer	3.6	2.6	3.1	
Comorbidities				
Congestive heart failure	7.2	6.5	5.3	.006
Diabetes	24.3	21.7	20.4	.004
Peripheral vascular disease	6.4	7.3	6.6	.38
Pulmonary circulation disorders	2.8	1.8	1.8	.06
Cardiac arrhythmias	22.1	20.6	22.1	.22
Chronic pulmonary disease	47.7	50.3	43.6	<.001
Liver disease	3.6	3.3	3.3	.88
Coagulopathy	3.8	2.7	2.4	.02
Neurologic disorders	4.0	3.0	3.3	.25

Data are reported as proportions or median (interquartile range). Statistical significance was set at α = 0.05.

HVH, high-volume hospital; LVH, low-volume hospital; MVH, medium-volume hospital; RATS, robotic-assisted thoracoscopic surgery; VATS, video-assisted thoracoscopic surgery.

### Morbidity and Mortality Analysis

After comprehensive risk adjustment, care at HVHs remained associated with significantly reduced likelihood of any major complication, with LVHs as reference (AOR, 0.81; 95% CI, 0.68-0.97; *P* = .02) (Figure). The difference in in-hospital mortality was similar (AOR, 0.99; 95% CI, 0.54-1.85; *P* = .99). With regards to specific complications, care at HVHs was linked with a lower likelihood of respiratory complications (AOR, 0.79; 95% CI, 0.65-0.96; *P* = .02) and a reduction in need for blood transfusion (AOR, 0.67; 95% CI, 0.48-0.93; *P* = .02). There was a nearly statistically significant decrease in the odds of infectious complications (AOR, 0.77; 95% CI, 0.57-1.02; *P* = .07).

**Figure.**
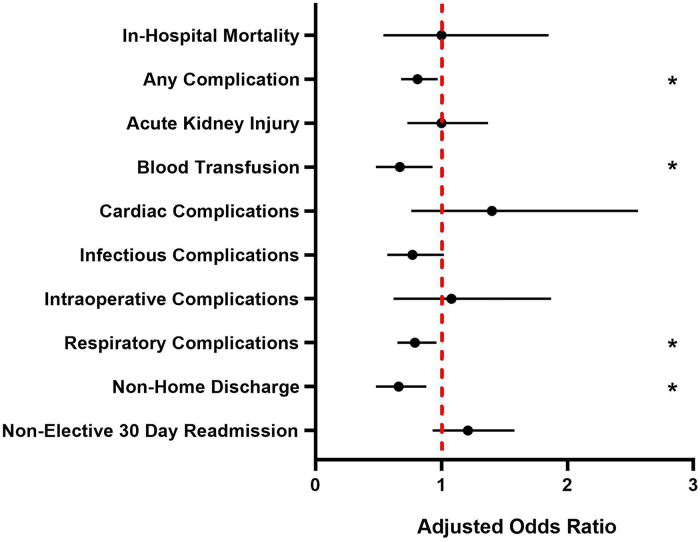
Risk-adjusted outcomes of high-volume and low-volume safety-net hospitals. The error bars represent 95% CI. ∗*P* < .05.

### Cost and Resource Utilization

Considering resource utilization, HVHs were associated with a reduced likelihood of nonhome discharge (AOR, 0.66; 95% CI, 0.48-0.88; *P* = .006), and a decrement in duration of hospitalization (β = −1.02 days; 95% CI, −1.48 to −0.54; *P* < .001) and overall expenditures (β = −$4360; 95% CI, −$7020 to −$1700; *P* = .001) (Table 2).

**Table 2:** Unadjusted and Adjusted Outcomes of Clinical Outcomes at Safety-Net Hospitals Compared With Non–Safety-Net Hospitals

Variable	Unadjusted			*P*	Adjusted		
	LVH	MVH	HVH		HVH	95% CI	*P*
Clinical outcomes							
In-hospital mortality	1.6	1.4	1.0	.04	1.00	0.54-1.85	.99
Any complication	31.1	23.8	21.3	<.001	0.81	0.68-0.97	.02
Acute kidney Injury	6.9	5.4	5.1	.05	1.00	0.73-1.37	.96
Blood transfusion	7.9	5.2	3.4	<.001	0.67	0.48-0.93	.02
Cardiac complications	1.4	1.5	1.4	.96	1.40	0.76-2.56	.28
Infectious complications	3.9	3.1	2.4	.005	0.77	0.57-1.02	.07
Intraoperative complications	2.1	1.5	1.6	.44	1.08	0.62-1.87	.79
Respiratory complications	21.8	16.1	14.3	<.001	0.79	0.65-0.96	.02
Nonhome discharge	8.3	7.5	4.9	<.001	0.66	0.48-0.88	.006
Nonelective 30-day readmission	6.4	7.3	6.9	.55	1.21	0.93-1.58	.16
Resource use							
Length of stay, d	6 (4-9)	5 (3-8)	4 (3-7)	<.001	−1.01	−1.48 to −0.54	<.001
Cost, USD $1000	30.6 (22.2-44.4)	27.4 (20.4-37.1)	27.5 (21.4-36.1)	<.001	−4.36	−7.02 to −1.70	.001

Unadjusted outcomes are reported as proportions or median (interquartile range). Adjusted outcomes are reported as the adjusted odds ratio with 95% CI, with LVH as the reference. Statistical significance was set at α = 0.05.

HVH, high-volume hospital; LVH, low-volume hospital; MVH, medium-volume hospital; USD, United States dollars.

## Comment

SNH status is associated with increased rates of perioperative complications and increased health care expenditures after pulmonary resection.^4^ Lung cancer resection is highly specialized and resource intensive. Limited data exist regarding the interplay between SNH and surgical volume. In this study, we found that surgical volume positively correlates with outcomes, specifically, less morbidity at SNH. Additionally, the difference in mortality was not statistically significant.

The benefit of surgical volume is well established in the literature. Maxwell and colleagues^5^ show that video-assisted thoracoscopic surgery volume is associated with decreased length of stay and decreased infection rates. In this study, we show that higher surgical volume correlates with decreased rates of any complications, respiratory complications, and nonhome discharges. The relationship between volume and outcomes has led many to advocate for centralization of care, most notably the Volume Pledge between 2015 and 2017, which advocated for restricting high-risk lung cancer resection to hospitals that met procedure-specific and surgeon volume criteria.^7^ However, centralization of care can significantly increase distance to care, particularly for those in rural or isolated areas, by causing lower-volume centers to close, thus imposing another significant barrier to care.^8^ Furthermore, volume alone does not guarantee good outcomes. Volume with validated quality improvement measures can portend better outcomes for patients on a larger scale. More work, particularly public health measures, is required to allow patients who frequent SNHs to benefit from opportunities that are available at non-SNHs.

Costs and expenditures are also important quality metrics. The relationship between surgical volume and costs varies in the literature. Maxwell and colleagues^5^ show an almost 20% reduction in costs to patients and the hospital system when lung resection is performed by a “high-volume” surgeon. On the other hand, a nationwide analysis of costs associated with major lung resection found no correlation between volume and costs.^9^

In this study, we find that overall expenditures at HVHs are lower compared with those at LVHs. Owing to the limitations of our database, whether this is associated with individual surgeon volume vs more efficient care pathways at a HVH is unclear. Furthermore, costs were likely focused on those related to the index hospitalization. Other factors that contribute to costs include stage at presentation, complexity of the case, and hospital policies for postoperative level of care.^10^ More work is required to determine what factors contribute to decreased costs at these high-volume centers. That being said, our findings do suggest a potential cost-saving opportunity for both patients and the hospital at high-volume centers.

### Limitations

The limitations of this study include those inherent to a retrospective database analysis, including reporting bias, selection bias, and missing data. We only analyzed patients undergoing lobectomy for lung cancer, and we did not analyze patients who underwent sublobar resections. Lobectomy care can be more nuanced with where coordinated care is necessary, which is why this was the focus for this study. Patients may also have been discharged with a chest tube in place to a Heimlich valve, which is not captured by this database. As mentioned before, our cost analysis likely included costs related to the index hospitalization and do not include travel cost for the patient and additional costs for readmissions. In addition, determining whether surgeons at any of these hospitals are thoracic-specific trained is difficult, which could affect outcomes. These variables are likely difficult to capture in a national database. Also as mentioned previously, we are unable to differentiate surgeon volume from hospital volume in a deidentified nationwide database.

### Conclusion

Surgical volume for pulmonary resection was associated with decreased perioperative complications, particularly respiratory complications, and costs at SNHs. Surgical volume did not impact mortality rates. These findings have implications for public health measures that can support patients traveling to higher-volume centers or systemically increase volume at low-volume centers for pulmonary resection.

## References

[1] Hoehn R.S., Wima K., Vestal M.A. (2016). Effect of hospital safety-net burden on cost and outcomes after surgery. JAMA Surg.

[2] Gilman M., Adams E.K., Hockenberry J.M., Milstein A.S., Wilson I.B., Becker E.R. (2015). Safety-net hospitals more likely than other hospitals to fare poorly under Medicare’s Value-Based Purchasing. Health Aff (Millwood).

[3] Wakeam E., Hevelone N.D., Maine R. (2014). Failure to rescue in safety-net hospitals. JAMA Surg.

[4] Sakowitz S., Verma A., Mabeza R.M. (2023). Clinical and financial outcomes of pulmonary resection for lung cancer in safety-net hospitals. J Thorac Cardiovasc Surg.

[5] Maxwell C.M., Bhat A.M., Falls S.J. (2024). Comprehensive value implications of surgeon volume for lung cancer surgery: use of an analytic framework within a regional health system. JTCVS Open.

[6] Agency for Healthcare Research and Quality Overview of the Nationwide Readmissions Database (NRD). https://hcup-us.ahrq.gov/nrdoverview.jsp.

[7] Urbach D.R. (2015). Pledging to eliminate low-volume surgery. N Engl J Med.

[8] Herb J., Dunham L., Stitzenberg K. (2019). Lung cancer surgical centralization disproportionally worsens travel burden for rural patients. J Am Coll Surg.

[9] Wakeam E., Hyder J.A., Lipsitz S.R., Darling G.E., Finlayson S.R.G. (2015). Outcomes and costs for major lung resection in the United States: which patients benefit most from high-volume referral?. Ann Thorac Surg.

[10] Cowper P.A., Feng L., Kosinski A.S. (2021). Initial and longitudinal cost of surgical resection for lung cancer. Ann Thorac Surg.

